# The Clinical Study on Short-Term Efficacy of Pelvic Magnetic Stimulation Combined with Pelvic Muscle Biofeedback on Female Idiopathic Overactive Bladder

**DOI:** 10.26502/fjwhd.2644-288400105

**Published:** 2023-03-24

**Authors:** LIU Xiaofang, LIN Fang, LUO Shuang, LU Zongjie, HE Quanjiang, MU Yan, PENG Qiao

**Affiliations:** 1 Suining central Hospital, Sichuan, Suining 629000, China

**Keywords:** Biofeedback, Magnetic Stimulation, Overactive Bladder

## Abstract

**Objective:**

To evaluate the short-term efficacy of pelvic magnetic stimulation combined with pelvic muscle biofeedback on female Idiopathic Overactive Bladder (IOAB).

**Methods:**

96 cases of IOAB females were randomly divided into control group (magnetic stimulation treatment n=48) and observation group (magnetic stimulation with biofeedback n=48). All the patients were dealt with the sacralneuromagnetic stimulation (once, qod), with 5 times in total. Then the patients in observation group were processed with biofeedback (10 times). Overactive Bladder Symptom Score (OABSS), Patients Perception Bladder Condition (PPBC) and Incontinence Quality of Life Scale (I-QOL) were evaluated as the indexes.

**Results:**

The effective rate of control group and observation group respectively were 89.58% and 93.75%. There were significant differences (P=0.67). The OABSS and PPBC of two groups were decreased, I-QOL were increased after treatment (P<0.05). Difference was statistically significant in OABSS three months after treatment between the two groups (P=0.00).The recurrence rate of three months after treatment of the two groups were 18.75% and 6.38% (P=0.04).

**Conclusions:**

Both magnetic stimulation alone and magnetic stimulation with biofeedback were effective and safe in female patients with IOAB. Magnetic stimulation with biofeedback may reduce the recurrence rate and continue to improve the symptoms in a certain extent.

Overactive bladder (OAB) is a syndrome characterized by symptoms of urinary urgency, often with urinary frequency and nocturia, and may be accompanied by urge incontinence [1, 2]. Its urodynamics are often characterized by overactivity of the bladder's forced urinary muscles. The etiology of OAB is unknown, and those with a disease duration of more than six months are called idiopathic OAB, while those with a clear etiology are called secondary OAB. The incidence of OAB is higher in women than in men, and men with OAB are often associated with prostate disease. Furthermore, pelvic floor muscle dysfunction is a particular risk factor for OAB in women, and the causes of OAB in women include overactivity of detrusor muscle and oversensitivity of bladder. Treatments advocated by the International Advisory Committee on Incontinence include behavioral therapy, pharmacotherapy, pelvic floor muscle biofeedback, and magnetic electrical stimulation. However, single-means therapies may have limitations. Single behavioral therapy requires patients to master the correct method and operate consistently. Single-drug therapy is prone to drug tolerance and cannot be applied long-term; pelvic floor muscle biofeedback is difficult to improve OAB caused by overactivity of the detrusor muscle. Magnetic stimulation is an in vitro neuromodulation technique, and magnetic stimulation at the S2-S4S level has been reported to inhibit excessive contraction of the detrusor muscle [3] , but the long-term efficacy needs further validation. As a result, magnetic stimulation and biofeedback training are different therapeutic targets and treatment modalities, and this prospective randomized controlled study was used to observe the short-term efficacy and safety of magnetic stimulation combined with pelvic floor muscle biofeedback.

## 1. Materials and Methods

### 1.1 Clinical Data

Ninety-eight patients with OAB treated at our pelvic floor rehabilitation center from January 2019 to January 2020 were grouped by complete randomization using the random number table method according to the inclusion and exclusion criteria. They were divided into control group (magnetic stimulation alone n=48) and observation group (magnetic stimulation combined with biofeedback n=48). (Ethical review approval number: 2016-15)

### 1.2 Inclusion and Exclusion Criteria

#### 1.2.1 Inclusion Criteria:

(1) women over 20 years of age; (2) disease duration of more than 6 months; (3) no relevant medication or physical therapy in the three months prior to being enrolled in this study (4) patients diagnosed with idiopathic OAB according to the International Association of Urological Control guidelines; (5) willing to cooperate to complete treatment and follow-up.

#### 1.2.2 Exclusion Criteria:

(1) those with moderate or higher pelvic organ prolapse; (2) patients diagnosed with secondary OAB; (3) those with metal or electronic devices in the body; (4) those with severe heart, liver, or kidney disease. (5) those with a history of epilepsy or Parkinson's disease.

### 1.3 Apparatus and Methods

#### 1.3.1 Pelvic Floor Magnetic Stimulation:

The pelvic floor magnetic stimulation equipment was the MS 080 magnetic stimulator (6.0 Tesla, 80 Hz) from Wuhan Ossef Medical Technology Co., Ltd. During treatment, the patient was placed in a lithotomy position so that the magnetic field of the electromagnetic coil could stimulate the sacrococcygeal nerve. The stimulation intensity was adjusted to just induce the S3 neuroreflex response of bilateral hip wind-like movement and foot toe flexion reflex, the stimulation frequency was 20 Hz, and the single treatment was 4200 pulses for 20 min once every other day.

#### 1.3.2 Pelvic Floor Muscle Biofeedback Treatment:

After the end of magnetic stimulation treatment in the observation group patients, the B4plus biostimulation counter was sequentially applied to the pelvic floor muscle biofeedback training by Nanjing McLand Medical Technology Co. Patients were instructed to maintain a semi-recumbent position with both lower limbs slightly abducted and relaxed, vaginal electrodes were inserted, and patients were instructed to contract and relax according to the treatment plan. The patient was instructed to perform contraction and relaxation training according to the treatment plan with 2-3 times per week, and 10 times in total.

### 1.4 Observation Indexes

#### 1.4.1 Quantitative Indicators:

The Overactive Bladder Scale (OABSS) was used as a quantitative diagnostic tool [4]. Patients with urinary urgency symptoms of 2 or more points and an overall OABSS score >3 were diagnosed with OAB; OAB patients were graded for severity: scores ≤5 mild, 6-11 moderate, ≥12 severe. The Patient Perception of Bladder Condition PPBC (PPBC) questionnaire assessed changes in OAB symptoms [5]. Incontinence Quality of Life Questionnaire (I-QOL) was used to assess the quality of life of patients [6].

#### 1.4.2 Clinical Efficacy Indexes:

**Cured**: the patient's symptoms of urinary frequency, urinary urgency and urinary incontinence disappears (the number of urination <8 times in 24h, nocturia <2 times, urine volume >200ml each time). **Apparent Effect:** the patient's symptoms of urinary frequency and urgency are relieved, and the number of urinary incontinence is reduced by 50%. **Ineffective:** patients had no relief of symptoms such as urinary frequency and urgency and no reduction in the number of urinary incontinence. Total effective rate = (cured + apparent effect)/total number of cases.

#### 1.4.3 Follow-up:

the patients in both groups are followed up by OABSS 3 months after treatment.

### 1.5 Statistical Methods

SPSS17.0 statistical software was applied for analysis and processing, and the measurement data were expressed as (±s) with t-test or paired t-test. Rates were expressed as percentages (%), and rates were compared using the pearson chi-square test, and the analysis of pre- and post-treatment and follow-up of OABSS in the two groups was performed using SNK ANOVA for comparison between groups and within groups before and after treatment. Differences were considered statistically significant at P < 0.05.

## 2. Results

### 2.1 General Information of the Two Groups

There was no statistically significant difference between the two groups in terms of age, pregnancy, delivery and mode of delivery (P > 0.05). (Table 1)

### 2.2 Follow-up Results

There were 96 cases in total. In the observation group, 1 case was terminated due to leaving the treatment place in the middle of the treatment, and the remaining 95 cases completed the treatment and follow-up, and the lost rate was 1%. During the treatment period, one case complained of dizziness after treatment, but the rest did not have any adverse reactions, and the average follow-up time was 3 months.

### 2.3 Qualitative Efficacy of Two Groups

In the control group, 16 cases were cured, 27 cases had significant effect and 5 cases had no effect among 48 patients, with an effective rate of 89.58%. Among 47 patients in the observation group, 19 were cured, 26 were effective, and 2 were invalid, with an effective rate of 93.75%. There was no statistical difference in the treatment efficiency between the two groups (P=0.67).

### 2.4 Observation of Quantitative Indexes in the Two Groups

The three indexes of OABSS, PPBC and I-QOL were compared between the two groups of patients before and after treatment (Table 2). The results showed that there was no statistically significant difference between the three indexes of OABSS, PPBC, and I-QOL before treatment between the two groups (P > 0.05); the OABSS score decreased from (9.50 ± 2.56) to (7.32 ± 1.67), PPBC decreased from (4.96 ± 1.12) to (2.13 ± 0.72), and I-QOL decreased from ( 18.80±15.37) to (34.40±12.24), with statistically significant differences (P<0.05). In the observation group, OABSS score decreased from (9.41±2.38) to (7.59±1.47), PPBC decreased from (4.85±1.03) to (1.80±0.75), and I-QOL increased from (20.86± 14.40) to (38.25±13.01), with a statistically significant difference (P<0.05). Using the SNK method, an ANOVA was performed for two-way comparison of OABSS before and after treatment in both groups, and there was a statistically significant difference between the two groups in OABSS scores before treatment, at the end of treatment and at 3 months after treatment (P=0.00). (Table 3)

### 2.5 Comparison of Recurrence Rates between the Two Groups

Patients were evaluated by OABSS 3 months after treatment in both groups, with 9 recurrence cases in the control group and 3 recurrence cases in the observation group, the recurrence rates in the two groups were 18.75% and 6.38%, respectively, and the difference was statistically significant (P=0.04).

## 3. Discussion

The pelvic floor musculature, nerves, and fascia form the overall structure of the female pelvic floor, which maintains urination, defecation, and sexual function. Once the overall structure is damaged, it may lead to the development of pelvic floor dysfunctional disorders. Overactive bladder (OAB), on the other hand, is a common abnormality in urinary function, and aging, changes in hormone levels in women, pregnancy and childbirth, and pelvic surgery can exacerbate OAB symptoms [7]. Because the etiology of idiopathic OAB is unknown, the current treatment goal is to improve clinical symptoms, and commonly used noninvasive treatments include pharmacotherapy, behavioral therapy, and Chinese medicine [8, 9,10], but the side effects of long-term pharmacotherapy seriously reduce patients' medication compliance [11]. In the face of patients with refractory OAB, clinicians consider invasive means such as forced urinary muscle toxin injection, sacral nerve electrical stimulation, and tibial nerve electrical stimulation, so as to inhibit nerve reflexes and suppress forced urinary muscle effects [12,13]. As the population ages and people's standard of living improves, more and more female patients want a long-term tolerable and non-invasive way to relieve their symptoms and improve their quality of life [14]. Electrical stimulation or magnetic stimulation has been used clinically to treat patients with OAB and has achieved certain results. Overactive bladder disorder is often associated with instability of the detrusor muscle and increased sensitivity of afferent nerve fibers, and extracorporeal magnetic stimulation uses a high intensity pulsed magnetic field outside the body to stimulate the nerves associated with the pelvic floor innervation, increase the mucosal M receptors at the nerve endings, inhibit the overactivity of the detrusor muscle, and improve the function of the innervated muscles and organs thereby achieving a therapeutic effect [15, 16,17]. Some foreign scholars have proposed that the effect of magnetic stimulation to inhibit the detrusor muscle by stimulating the sacral nerve roots is significantly stronger than that of electrical stimulation [18], which may be related to the physical properties of electrical stimulation, where the current density is highest where the electrodes are in close contact and decays rapidly with the depth of the tissue, and the effect is correspondingly weakened. In 2007, CHOE proposed that the cure rate of patients with OAB treated with magnetic stimulation alone after 2 weeks of treatment could reach 56.3% [19]. In this study, magnetic stimulation combined with biofeedback was observed for the first time in comparison with magnetic stimulation alone, and the results showed that both magnetic stimulation alone and magnetic stimulation combined with biofeedback were significantly effective in the treatment of idiopathic OAB, with an effective rate of 89.58% and 93.75%, respectively, with no statistically significant difference between the two groups (P=0.67). The cure rate of magnetic stimulation alone was 33.33%, which was lower than the results of the CHOE study and may be related to the shorter treatment course in this study. The OABSS, PPBC, and I-QOL indexes were significantly improved at the end of treatment in both groups compared with those before treatment, and the OABSS index was still better than that before treatment at 3 months after treatment (P < 0.05). The difference in OABSS between the two groups at 3 months after treatment was statistically significant (P=0.00), and in terms of the relapse rate at 3 months, the relapse rate in the two groups was 18.75% versus 6.38%, respectively, with a statistically significant difference (P=0.04), indicating that in the short term, magnetic stimulation combined with biofeedback treatment can reduce the relapse rate to a certain extent and is more conducive to sustained symptom improvement.

The present study and related domestic and international studies confirmed that magnetic stimulation was effective in the treatment of OAB, and that extracorporeal magnetic stimulation combined with biofeedback can be the first choice for the clinical treatment of idiopathic OAB because of its non-invasive, safe, and patient-friendly characteristics. Since the follow-up period of this study was relatively short and there were differences in individual behavioral patterns of patients, a larger sample and long-term follow-up are needed to confirm the effectiveness of magnetic stimulation combined with biofeedback on the sustained improvement of symptoms and the recurrence rate of OAB patients.

## Figures and Tables

**Table 1:** Comparison of the general conditions of the two groups.

Group	Age	History of childbirth		Different mode of delivery	
		Gravida	Times of childbirth	Eutocia	Cesarean delivery
Control group	42.23±9.44	3.60±0.58	2.42±0.62	26	22
Observation group	41.18±9.47	3.84±0.47	2.27±0.34	23	25

**Table 2:** Comparison of observed indicators before and after treatment between two groups of OAB patients ($\bar{\chi }\pm s$).

Group	OABSS			PPBC			I-QOL		
	Before treatment	After treatment	P- value	Before treatment	After treatment	P- value	Before treatment	After treatment	P-value
Control group	9.50±2.56	7.32±1.67	0	4.96±1.12	2.13±0.72	0.01	18.80±15.37	34.40±12.24	0
Observation group	9.41±2.38	7.59±1.47	0	4.85±1.03	1.80±0.75	0.02	20.86±14.40	38.25±13.01	0

Notes: OABSS- Overactive Bladder Symptom Score; PPBC- Patient Perception of Bladder Status Questionnaire; I-QOL- Quality of Life Questionnaire for Urinary Incontinence.

**Table 3:** Comparison of OABSS scores between and within groups at 3 months after treatment in both groups $\bar{\chi }\pm s$.

Group	Before treatment	End of treatment	At 3 months after treatment	F-value	*P-value*
Control group	9.50±2.56	7.32±1.67	8.95±1.79	7.217	0
Observation group	9.41±2.38	7.59±1.47	7.59±1.56	7.951	0

Note: Analysis of variance between and within groups by SNK method
